# Double gene mutations of LRSAM1 and REEP1 and a new REEP1 mutation site found in a patient with amyotrophic lateral sclerosis with subjective paresthesia: A case report

**DOI:** 10.1002/ibra.12125

**Published:** 2023-08-14

**Authors:** Ji‐Yao Qin, Zun‐Lin Zhou, Yuan‐Qing Zhou, Ya Chen, Xiao‐Yan Yang, Hai‐Qing Zhang, Zu‐Cai Xu

**Affiliations:** ^1^ Department of Neurology Affiliated Hospital of Zunyi Medical University Zunyi Guizhou China

**Keywords:** amyotrophic lateral sclerosis, hereditary spastic paraplegia (SPG31), *LRSAM1*, peroneal muscular atrophy type 2p, receptor expression enhancing protein 1

## Abstract

Amyotrophic lateral sclerosis (ALS) is a neurodegenerative disease characterized by selective degeneration of upper and lower motor neurons. Although dyskinesia is the most prominent clinical manifestation of ALS, with an in‐depth understanding of disease pathogenesis and clinical detection, more and more ALS patients are found to have nonmotor symptoms, such as sensory impairment. Genetic testing technology has developed rapidly in recent years. New genes have been proven to be involved in the pathogenesis of ALS. However, according to the existing research evidence, no literature has reported that patients with ALS have leucine‐rich repeats and sterility α mutations in motif 1 (*LRSAM1*) and receptor expression accessory protein 1 (*REEP1*). The mutation sites of *REEP1* gene have not been reported, and the simultaneous mutations of two genes have not been reported. In the largest human gene mutation frequency database gnomad, the mutation sites of two genes are currently defined as new heterozygous variants with unclear clinical significance. Therefore, this article reports the clinical data of this case to further deepen the clinicians' understanding of the disease, and may provide evidence for further study of the new genotype–phenotype of *LRSAM1* and *REEP1*.

## Graphical abstract

Amyotrophic lateral sclerosis (ALS) is a neurodegenerative disease characterized by selective degeneration of upper and lower motor neurons. Leucine‐rich repeats and sterility α mutations in motif 1 (LRSAM1) has been proven to be involved in the pathogenesis of ALS. LRSAM1 is an E3 ubiquitin ligase and is involved in the cellular protein quality control of neurons. When LRSAM1 is mutated, which may interfere with E3 ligase activity, LRSAM1 becomes impaired in clearing the misfolded protein. Nascent polypeptide chains are under constant threat of misfolding from various stresses and misfolded polypeptides are highly susceptible to aggregate formation in neurons. Subsequently, neurological dysfunction may occur.

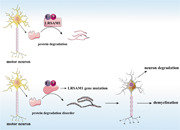

## 1 INTRODUCTION

Amyotrophic lateral sclerosis (ALS) is a fatal neurodegenerative disease characterized by selective involvement and degeneration of upper and lower motor neurons. Most patients have sporadic ALS (sALS) and about 10% have familial ALS.^1^ For a long time, it was believed that ALS was a purely motor‐involving degenerative disease that did not involve the sensory neurons of the dorsal root ganglion. The sensory nerve conduction in ALS patients is usually normal. And if there is damage, it can basically rule out the disease.^2^ However, in recent years, with the advancement of medical technology, new detection technologies, and methods have been applied to the clinic.^3^ And it has been proved that ALS patients also have sensory nerve dysfunction, making the view that “ALS can also be accompanied by sensory nerve involvement” increasing.^4^ At present, the methods of detecting sensory disturbance in sALS include neuroelectrophysiology, neuropathology, neuroimaging, animal model simulation, and so forth. And some studies have shown that sALS complicated with sensory disturbance, has a certain genetic predisposition. However, the results obtained by different teams and different research methods are inconsistent. In neurophysiological examinations, 15%–90% of patients with ALS are found to have paresthesias.^5^ The results of the diffusion tensor imaging (DTI) study by Iglesias et al.^6^ showed that about 60% of patients had anatomical damage to ascending sensory fibers. Corneal confocal microscopy (CCM) is an imaging technique developed in recent years, which can observe small corneal sensory fibers in vivo. Examination of a cohort of sALS patients using CCM showed a reduction in the number and branches of corneal fibrillar sensory nerves in sALS patients.^7^ According to statistics, about 5%–30% of ALS patients have sensory symptoms and signs. Among these symptoms, numbness is the most common symptom, followed by decreased pain and temperature sensation. Among the paresthesia signs, the most common is vibrational hypoesthesia.^6^, ^8^ The mechanism of ALS remains unclear, and most cases are sporadic, but genetic factors may play an important role. More than 20 genes have been reported to be associated with ALS. The five most common genetic causes are hexanucleotide amplification of Chromosome 9 open reading frame 72 and superoxide dismutase 1 (*SOD1*), *TAR* DNA binding protein 43, fusion sarcoma, *TANK*‐binding kinase 1 mutations.^9^ However, there is no report in the literature that leucine‐rich repeats and sterility α mutations in motif 1 (*LRSAM1*) and receptor expression accessory protein 1 (*REEP1*) are related to ALS. The simultaneous mutation of both *LRSAM1* and *REEP1* genes has not been reported in the literature; the gene mutation of *REEP1* is a new mutation site, and the mutation sites of *LRSAM1* and *REEP1* are mutations of unknown clinical significance. This case reported a female patient with ALS with subjective sensory anomalies, through genetic testing and other methods, found that the patient's pathogenesis may be due to double gene mutations in *LRSAM1* and *REEP1*, and a new *REEP1* mutation site. Therefore, in addition to expanding the knowledge of *LRSAM1* and *REEP1* gene mutation sites, this case can also provide a reference for genetic counseling and prenatal diagnosis and is of great help to study the pathogenesis of the disease.

## 2 CASE DESCRIPTION

This case report was approved by the ethics committee of the Affiliated Hospital of Zunyi Medical University on February 15, 2023 (approval no: KLL‐2023‐022).

**Figure 1:**
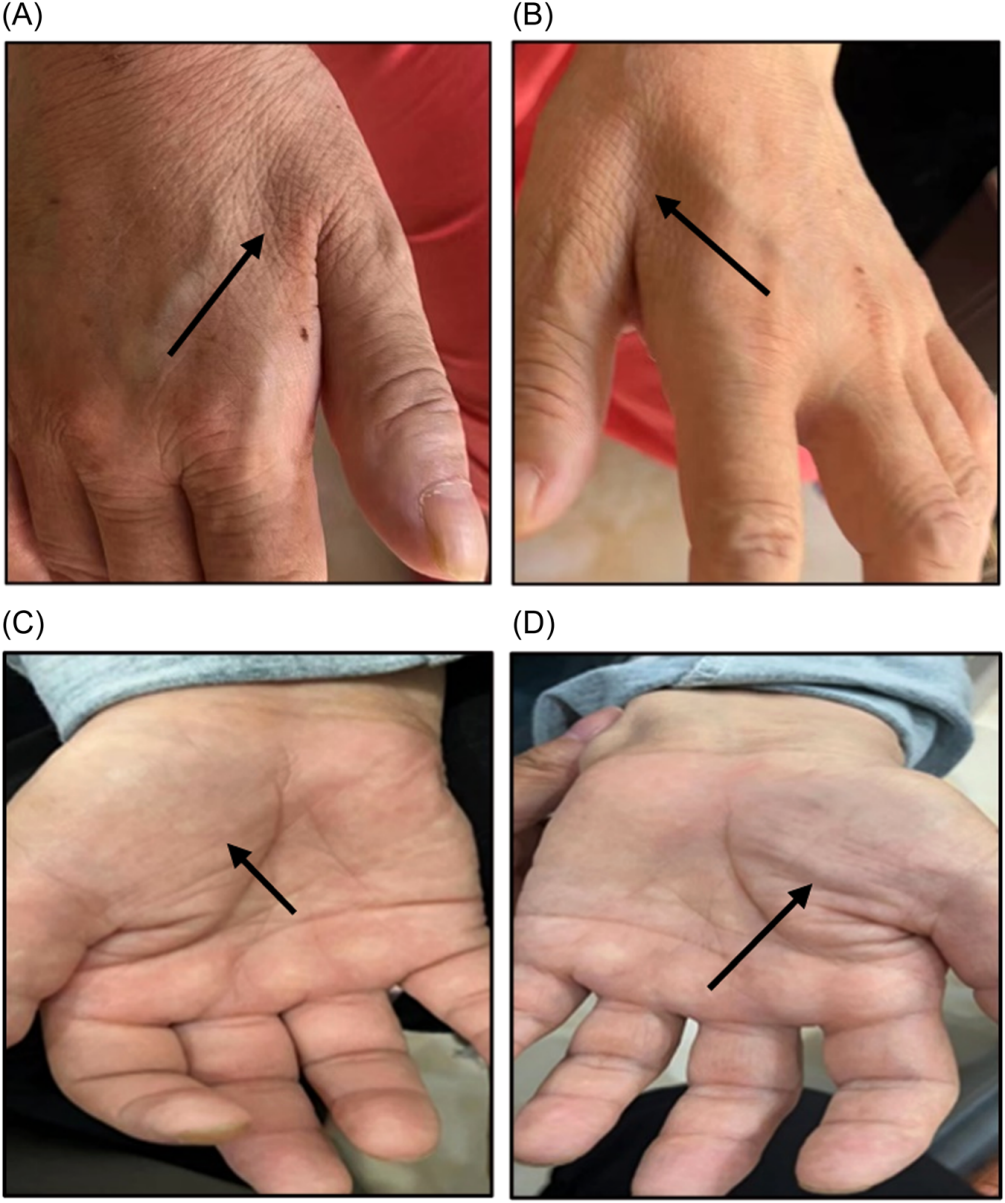
Palmar and dorsal interossei muscle atrophy of both hands. The arrows in the figure show atrophy of the interosseous muscles in the back of the palms of the patient's hands. The arrow in (A) shows the atrophy of the first interosseous dorsal muscle of the right hand. The arrow in (B) shows the atrophy of the dorsal muscle of the first interosseous muscle of the left hand. The arrow in (C) shows the atrophy in right‐hand thenar muscle. (D) shows the atrophy of muscle of thenar in the left hand. [Color figure can be viewed at wileyonlinelibrary.com]

**Figure 2:**
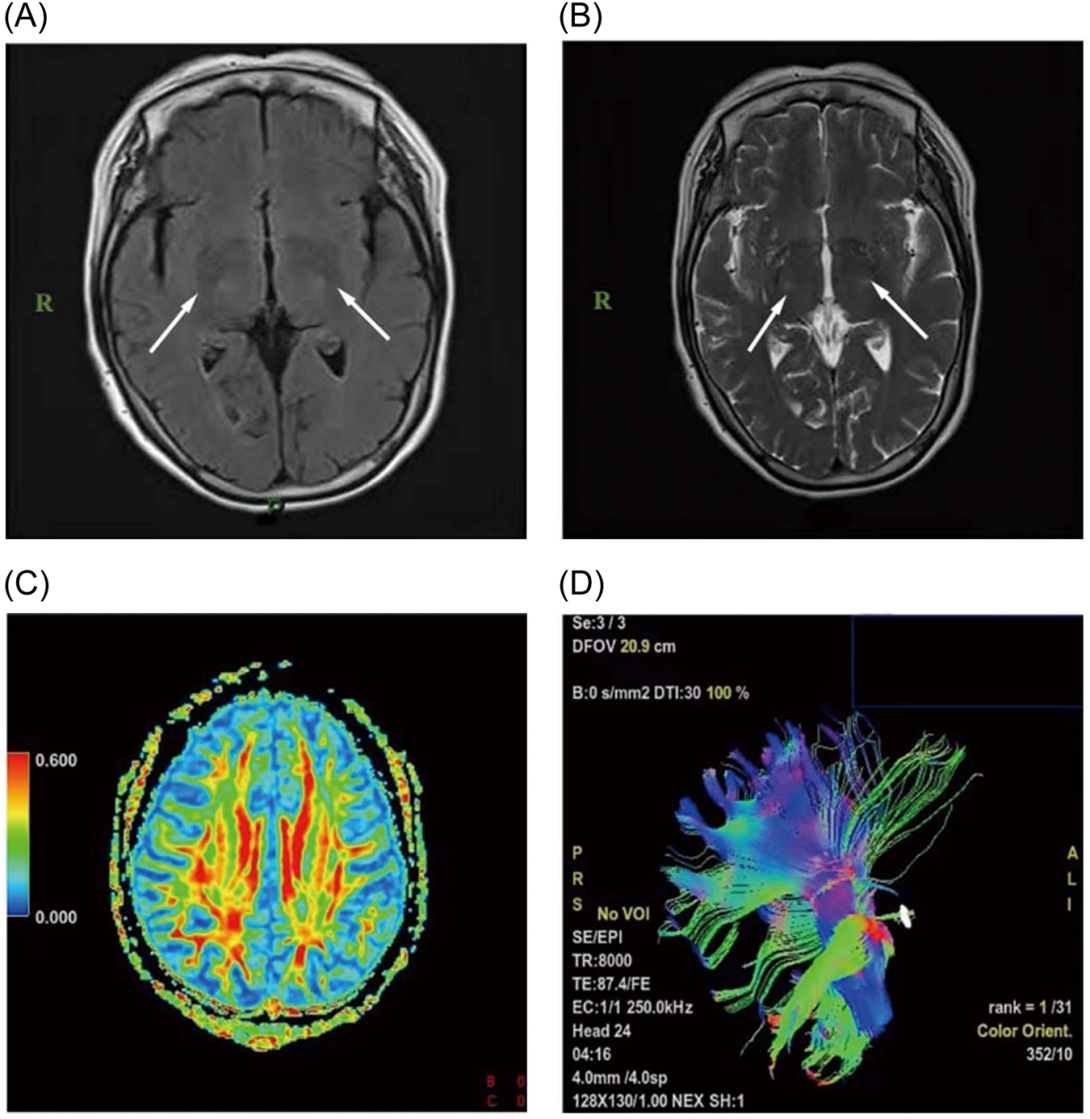
Brain magnetic resonance imaging. (A) T1‐weighted imaging. (B) T2‐weighted imaging. The arrows in (A) and (B) show bilateral corticospinal tract abnormalities. T Panels (C) and (D) are brain MRI nerve fiber bundle angiography, and no abnormal manifestations were shown in the figure. MRI, magnetic resonance imaging. [Color figure can be viewed at wileyonlinelibrary.com] [Correction added on 24 July 2025, after first online publication: The figure 2 was revised to improve the clarity by adding the arrows.]

A 57‐year‐old female patient developed right upper limb weakness without obvious incentive half a month ago and was admitted to in the Department of Neurology on March 4, 2022. Her right calf muscle spasm began without obvious incentive half a month ago, mainly at night, and right upper limb weakness occurred more than 10 days ago with neck, shoulder, and right upper arm pain. At the same time, on the basis of the right calf muscle spasm that mainly occurred at night, weakness in the right lower extremity began to appear. In March 2022, she was admitted to the Department of Neurology, Affiliated Hospital of Zunyi Medical University. The patient was diagnosed with tenosynovitis due to a mass in the right wrist 5 years ago, and the patient improved after surgical treatment. She denied a similar medical history of family members and inherited diseases or genetic predispositions in two and three generations. The patient's parents have passed away, and the genetic test results of her two daughters and one son are normal. The patient's five siblings all refused genetic testing because they had no symptoms such as limb weakness. Physical examination: Vital signs were stable and no abnormality was found in the general medical system. Nervous system physical examination: palmar and dorsal interossei muscle atrophy of both hands (Figure 1), no muscle atrophy in the limbs, muscle strength of the right upper extremity Grade 3, muscle strength of the right lower extremity Grade 4, hyperreflexia of the limbs, bilateral ankle clonus and patella positive clonus, positive bilateral Lotholimo sign, bilateral Hoffman's sign was positive, and physical examinations such as sensation were normal. Examination: There were no obvious abnormalities in the test results of three items of anemia, blood calcium, and vitamin D. Cerebrospinal fluid protein quantification: 529 mg/L, cerebrospinal fluid white blood cell count: 9 × 10^6^/L, and other indicators are normal. Cervical, thoracic, and lumbar magnetic resonance imaging (MRI) showed mild degeneration and no nerve root and spinal cord compression. Brain MRI showed abnormal signals in bilateral corticospinal tracts (Figure 2A,B). Brain MRI nerve fiber tractography (DTI): no obvious abnormality (Figure 2C,D). Examination of nerve function: neurogenic damage of limbs (multiple cervical, lumbar, and sacral nerve root damage is possible); the source of the anterior horn of the spinal cord cannot be removed, and the right sternocleidomastoid muscle, seventh thoracic vertebrae (T7) and tenth thoracic vertebrae (T10) paraspinal muscles show spontaneous electrical activity (anterior spinal cord angle and brainstem motor nerve sources) (Table 1). The results of genetic testing indicated that the c.913C>T (p.Arg305Trp) variant carried by the patient was a missense variant in the coding region of the *LRSAM1* gene, and the c.182+8C>G variant was a minor variant in the intron region of the *REEP1* gene. The patient was considered for ALS (with subjective paresthesias). During hospitalization, symptomatic treatments such as brain strengthening and circulation improvement were given. After 3 months of follow‐up, it was found that the patient gradually developed left limb weakness on the basis of the weakness of the right limb. In the case of serious situations in the upper limbs, the right limb weakness was more serious than the left limb. With the pain in the right upper limb, no muscle spasm in the right lower limb, and problems in chewing or swallowing, the right limb was more serious than the left limb.

**Table 1:** Resting potential by needle electromyography.

Muscle	Positive sharp wave	Fibrillation	Tremor	Twitch
Left seventh thoracic vertebrae (T7) paraspinal muscle	−	−	−	−
Right third thoracic vertebrae (T3) paraspinal muscle	−	−	−	−
Right tenth thoracic vertebrae (T10) paraspinal muscle	+	+	−	−
Right seventh thoracic vertebrae (T7) paraspinal muscle	+	−	−	−
Right sternocleidomastoid muscle	−	2+	−	−

*Note*: The table above shows the results of the patient's needle‐type electromyography determination of resting potential. “+” Represents a positive signal, while “−” represents a negative signal. The patient's right paravertebral tenth muscle showed positive signals on positive sharp waves and myofiber tremor. At the same time, the right seventh paravertebral muscle also showed a positive signal on positive sharp waves. The patient's right sternocleidomastoid muscle also showed a strong positive signal on muscle fiber tremor.

## 3 DISCUSSION

ALS is a progressive and fatal motor neuron neurodegenerative disease for which there is currently no effective treatment. It is widely believed that genetic predisposition, aging, and environmental exposure are associated with ALS, involving several cellular processes and mechanisms in protein aggregation and misfolding, oxidative stress, mitochondrial dysfunction, and glutamate excitotoxicity. In recent research, transport defects, energy exhaustion, and dysregulation of intracellular Ca^2+^ homeostasis, are also involved in the pathogenesis of ALS.^10^ Since the discovery of the first ALS pathogenic gene *SOD1* in 1993, the etiology and genetics of ALS have progressed rapidly. More and more ALS pathogenic genes have been discovered one after another, but whether *LRSAM1* and *REEP1* are related to ALS is still unclear. *LRSAM1*, encoding the E3 ubiquitin ligase, is a very interesting new gene (loop) class of proteins.^11^ E3 ubiquitin ligases constitute a large family of enzymes that can modify their target proteins through covalent attachment of ubiquitin molecules, which will inhibit the accumulation of misfolded protein aggregates and promote protein aggregate degradation, and mitigate their harmful effects cytotoxicity.^12^ *LRSAM1*‐related mutations are located at the boundary of the C‐terminal ring finger domain, which may interfere with E3 ligase activity, thereby sensitizing peripheral axons to degeneration.^13^ Aggregation of protein aggregates is a key feature of neurodegenerative disease pathology. The neuropathology of ALS also found the formation of protein aggregates such as TDP‐43.^14^ *LRSAM1* encodes an E3 ubiquitin ligase. E3 ubiquitin ligases can modify their target proteins through covalent attachment of ubiquitin molecules to inhibit the accumulation of misfolded protein aggregates and promote their degradation, and mitigate their deleterious cytotoxic effects.^14^ Whether *LRSAM1* c.913>T (p.Arg305Trp) is a pathogenic mutant, and whether it is related to ALS and participates in the pathogenesis of ALS by removing protein aggregates deserves further study.

With the continuous development of genetic testing technology, more and more genes have been confirmed to be related to the pathogenesis of motor neuron disease. However, there is no report in the literature that *REEP1* is associated with ALS. The genetic testing of the ALS patients we treated found *REEP1* c.182+8C>G, and this mutation was not reported in the two most comprehensive and authoritative gene databases, Human Gene Mutation Database and gnomAD. REEP1 belongs to a transmembrane protein of the TB2/HVA22 family.^15^ Although the *REEP1* c.182+8C>G mutation site is located in the intron region, the intron will be spliced out after the gene is transcribed into precursor RNA to become mature RNA. However, the intron has the recombination of exons in the genome Intronic mutations and splicing abnormalities are also pathogenic due to their ability to form new genes.^16^ Mitochondria‐associated endoplasmic reticulum membranes (MAMs) are dynamic membrane‐coupling regions formed by the coupling of mitochondrial outer membrane and endoplasmic reticulum. MAMs are involved in mitochondrial dynamics, mitochondrial phagocytosis, Ca^2+^ exchange, and endoplasmic reticulum stress.^17^ *REEP1* plays an important role in the structure and function of MAMs.^18^ The ALS mechanism includes abnormal MAMs.^19^ The significance of the *REEP1* c.182+8C>G mutation is unknown. Is it pathogenic? Is it related to ALS, and if so, what role does it play in it? These series of questions are unknown, and further basic and clinical research is needed to answer them.

In addition to considering whether *LRSAM1* and *REEP1* are related to ALS and participate in its pathogenesis, we can also consider whether this patient is comorbid with ALS and other diseases. REEP1 belongs to a transmembrane protein of the TB2/HVA22 family, and *REEP1* mutations include missense, nonsense, frameshift, and splice site deletions.^15^ Mutations in *REEP1* cause autosomal dominant hereditary spastic paraplegia (HSP) type SPG31. Previous studies have demonstrated distinct molecular pathogenesis of SPG31, including loss‐of‐function, gain‐of‐function, and haploinsufficiency.^20^ Recent studies have shown that *REEP1* mutation leads to endoplasmic reticulum stress deficiency and is involved in the pathogenesis of SPG31.^21^ The clinical manifestations of most patients with SPG31 are limited to lower extremity pyramidal tract signs, but some patients have complex phenotypes associated with peripheral axonopathy, and a few have cerebellar ataxia, tremor, and dementia.^22^, ^23^ The symptoms of the patients we treated were unilateral, and the upper extremity was the first symptom and the upper extremity symptoms were severe. There was no muscle spasm and obvious muscle atrophy at the follow‐up, which did not conform to the clinical manifestations of SPG31, although the electrophysiological and imaging examinations were consistent. Therefore, the comorbidity of SPG31 and ALS was not considered.

Mutations in *LRSAM1* have been identified as the genetic etiology of both dominant and recessive forms of Charcot‐Marie‐Tooth disease type 2P (CMT2P), and mutations have been identified as rare causes. CMT2P, an axonal type CMT (CMT2), has a clinical manifestation with typical symptoms of peripheral nerve deficits, such as slowly progressive distal muscle weakness and atrophy, with distal sensory loss, higher‐order gait, foot deformities, and diminished or absent tendon reflexes. Electrophysiological findings suggest decreased compound motor axon potentials. Since the *LRSAM1*‐related mutation in CMT2 is located at the boundary of the C‐terminal ring finger domain, it may interfere with E3 ligase activity. Loss of LRSAM1 function in CMT2P sensitizes peripheral axons to degeneration. In our case, the patient had obvious right upper extremity paresthesia and weakness. While the electrophysiology results suggested neurogenic damage, the symptoms were asymmetric. In fact, the patient had the behavior of central nervous system involvement, which did not meet the peripheral neuropathy CMT2P diagnosis. Although it is clear that *LRSAM1* c.913C>T (p.Arg305Trp) is related to CMT2P in the gnomAD database (http://gnomad-sg.org/), the clinical significance of this mutation site is unclear, and the possibility of comorbidity is not considered for the time being.

The clinical manifestations, imaging, and electrophysiology of the three diseases of ALS, HSP, and CMT overlap partially. Careful identification is essential for early diagnosis and treatment of the disease. Early diagnosis and genetic counseling can reduce the burden on patients and families. The high clinical and genetic heterogeneity of SPG31 and CMT2P makes diagnosis challenging. With the iteration of testing technology and the improvement of precision, genetic testing is still the gold standard for the diagnosis of both diseases. Although the relationship between SPG31 and *REEP1*, CMT2P and *LRSAM1* is almost a pairwise relationship, with the increasing inclusion of gene variation data in the human genetic disease gene database, the linear relationship between specific genes and specific diseases will also be impacted, especially for clinical significance. Whether the results of genetic testing for unknown gene mutations can be used as one of the criteria for disease diagnosis and differential diagnosis deserves careful consideration and further discussion.

Our case also has shortcomings in that the patient's parents died, and the patient's siblings did not cooperate to complete the genetic test. So, there was a lack of complete family genetic data.

## AUTHOR CONTRIBUTIONS

Ji‐Yao Qin, Zun‐Lin Zhou, Yuan‐Qing Zhou, and Hai‐Qing Zhang designed and wrote this article. Xiao‐Yan Yang, Ya Chen, and Zu‐Cai Xu helped with proofreading and revision. All authors read, revised, and approved the final manuscript.

## CONFLICT OF INTEREST STATEMENT

The authors declare no conflict of interest.

## TRANSPARENCY STATEMENT

The authors affirm that this manuscript is an honest, accurate, and transparent account of the study being reported; that no important aspects of the study have been omitted; and that any discrepancies from the study as planned (and, if relevant, registered) have been explained.

## ETHICS STATEMENT

This case report was approved by the ethics committee of the Affiliated Hospital of Zunyi Medical University (Approval No: KLL‐2023‐022). Written informed consent was obtained from the patient for publication of this case report. The patients also signed informed consent for the use of tissues in this study.

## Data Availability

The authors confirm that the data of this study are available within the article.
